# Multi-walled carbon nanotube-supported metal-doped ZnO nanoparticles and their photocatalytic property

**DOI:** 10.1007/s11051-012-1295-5

**Published:** 2012-12-16

**Authors:** C. S. Chen, T. G. Liu, L. W. Lin, X. D. Xie, X. H. Chen, Q. C. Liu, B. Liang, W. W. Yu, C. Y. Qiu

**Affiliations:** 1 College of Physics and Electronic Science, Changsha University of Science and Technology, Changsha, 410114 People’s Republic of China; 2 State Key Laboratory for Powder Metallurgy, Central South University, Changsha, 410083 People’s Republic of China; 3 College of Physics and Microelectronics Science Hunan University, Changsha, 410082 People’s Republic of China

**Keywords:** Carbon nanotube, Metal-doped ZnO, Oxide hybrid, Photocatalytic property

## Abstract

A simple and versatile approach has been developed to synthesize multi-walled carbon nanotubes/metal-doped ZnO nanohybrid materials (MWNT/M-doped ZnO) by means of the co-deposition method. The experimental results illuminate that MWNTs can be modified by metal-doped ZnO nanoparticles at 450 °C, such as Mn, Mg, and Co elements. Furthermore, the MWNT/Mg-doped ZnO hybrids have been proven to have a high photocatalytic ability for methyl orange (MO), in which the degraded rate for MO reaches 100 % in 60 min. The enhancement in photocatalytic activity is attributed to the excellent electriconal property of MWNTs and Mg-doping. The resultant MWNT/Mg-doped ZnO nanohybrids have potential applications in photocatalysis and environmental protection.

## Introduction

Carbon nanotubes (CNTs) have attracted considerable research interest in the last decade because of their unique optical, electronic, magnetic, mechanical, and gas adsorption properties. They have been regarded as promising candidates for versatile applications. Exhibiting high electrical conductivity and high electron storage capacity (one electron for every 32 carbon atoms) (Kongkanand and Kamat 2007; Kongkanand et al. 2007), CNTs can act as extremely effective electron sinks. Hence, CNTs supported with metal oxide nanoparticles are expected to exhibit different physical properties from those of neat CNTs.

Recently, CNTs have been used as templates or scaffolds for the hybrid assembly of nanoparticles, and are widely reported to synergistically enhance the photocatalytic activity of oxide nanoparticles through the retardation of electron–hole recombination (Leary and Westwood 2011; Woan et al. 2009; Vietmeyer et al. 2007). However, CNTs often exist in the form of highly tangled ropes and show poor solubility. Furthermore, it is very difficult to control the inhomogeneity and the quality of oxide nanoparticles coating on the surface of CNTs, which prevent their applications in various devices (Karousis and Tagmatarchis 2010). To efficiently synthesize CNT-based nanooxide hybrids, it is vital to activate the graphitic surface of CNTs.

Zinc oxide (ZnO) has become one of the promising candidates for the photocatalysis and degradation of various organic pollutants because of its high optical activity and stability, wide band gap of 3.37 eV, a large excitation-binding energy (60 meV) at room temperature, low cost, and environmental friendliness. Many approaches have been used to synthesize CNT–ZnO hybrids that possess good optical and photocatalytic properties (Eder 2010; Liu et al. 2006; Chen et al. 2006; Yang et al. 2002; Samadi et al. 2012; Jiang and Gao 2005; Zhu et al. 2009; Khataee and Zarei M. 2011; Saleh et al. 2011). In these approaches, CNTs or inorganic nanoparticles require modification with organic functional groups, and then inorganic nanoparticles are attached to the surface of CNTs via covalent, noncovalent, or electrostatic interactions. Our previous article reveals that MWNTs can directly be coated by Cu-doped ZnO nanoparticles through co-precipitation method after MWNTs are treated with sodium hydroxide and mixed acid (Chen et al. 2012). Moreover, the attained composite powders were proved to possess excellent optical property.

In this article, we further study MWNTs decorated other metal-doped ZnO nanoparticles. The mechanism of MWNT-supported nanoparticles is also analyzed. In addition, the photocatalytic property of MWNT/metal-doped ZnO hybrids is studied.

## Experimental

### Preparation and treatment of MWNTs

As-prepared MWNTs (diameters 20–50 nm) were prepared by the chemical catalytic vapor decomposition process (CVD). The details of the MWNTs preparation have been described in the literature (Chen et al. 2002). In a typical treatment, 5 g as-prepared MWNTs was dispersed in 500 mL sodium hydroxide solution (2 mol/L) and refluxed at boiling for 2 h under stirring. After being rinsed with deionized water until the pH value of solution close to neutral, the NaOH-treated MWNTs were dried at 80 °C. In order to remove impurities, these NaOH-treated MWNTs were further oxidized by immersing in a 3:1 mixture of concentrated H_2_SO_4_ and HNO_3_ and refluxing for 2 h at boiling point, subsequently suspending, and refluxing in HCl solution for 2 h at the same temperature. Finally, the MWNTs were dried at 80 °C after being filtered and washed with deionized water. For comparison, the as-prepared MWNTs were oxidized by immersing in a 3:1 mixture of concentrated H_2_SO_4_ and HNO_3_ and refluxing for 2 h at boiling point, subsequently suspending, and refluxing in HCl solution for 2 h at the same temperature.

### Preparation of MWNTs–inorganic hybrids

MWNT/Mn-doped ZnO nanohybrids were performed typically as follows: 2.2 g Zn*(*CH_3_COO*)*_2_·2H_2_O and Mn*(*CH_3_COO*)*_2_·2H_2_O (Mn/Zn = 5 % in molar ratio) were first dissolved in anhydrous ethanol of 100 mL, and then 0.05 g of the above-treated MWNTs was added into under sonicating for about 15 min. Subsequently, the mixture solution, which comprises oxalic acid and anhydrous ethanol of 100 mL, was slowly dropped into the mixture solution of zinc acetate and manganese acetate with stirring at 60 °C, and a sol was produced. Third, the sol was maintained at 80 °C for 48 h to form the precursor. Finally, the above-prepared precursor was annealed at 450 °C for 2 h under the protection of nitrogen.

MWNT/Mg-doped ZnO nanohybrids and MWNT/Co-doped ZnO nanohybrids were fabricated using similar procedures except for the replacement of Mn*(*CH_3_COO_2_ *)*·4H_2_O using Mg(CH_3_COO)_2_·4H_2_O, Co(CH_3_COO)_2_·4H_2_O, respectively. The molar ratio of Mg to Zn as well as Co to Zn is 1:9. For comparison, the ZnO nanoparticles and MWNT/ZnO composite powder annealed at 450 °C were prepared under the same conditions.

### Characterization

Scanning electron microscopy (SEM) observations were carried out using a JSM-6700F field emission scanning electron microscope. Transmission electron microscope (TEM) images, high-resolution transmission electron microscope (HRTEM), and energy dispersive X-ray spectroscopy (EDS) were performed using a JEM-3010 microscope on powder samples deposited onto a copper micro-grid coated with holey carbon. X-ray diffraction (XRD) measurements were performed using Philips PW 1710 diffractometer with Cu Kα_1_ radiation.

Infrared spectroscopy was performed using a 300E Jasco spectrophotometer at room temperature. The O *K*-edge X ray absorption fine structure (NEXAFS) measurement of MWNTs was performed using the soft x-ray magnetic circular dichroism (SXMCD) beamline with an electron beam (e beam) having energy range of 100–1,000 eV and an average beam current of 100–300 mA at the National Synchrotron Radiation Laboratory (NSRL), University of Science and Technology of China. The porous texture of MWNTs samples was analyzed by N_2_ adsorption at 77 K, and the surfaces areas were calculated by applying the BET equation to the N_2_ isotherm data. Fluorescence spectra measurements were characterized for anhydrous ethanol on a Hitachi F4500 fluorescence spectrophotometer at room temperature.

Methyl orange (MO) was used as a model dye to evaluate the photocatalytic activity of MWNT/metal-doped ZnO hybrids. In a typical experiment, 50 mg catalyst was dispersed in 200 mL of 20 mg/L MO aqueous solution. The above reaction mixture were conducted at room temperature under a highpressure Osram Ultra-Vitalux lamp (100 W, wave length 365 nm) positioned horizontally above the liquid surface (the lamp was placed at about 40 cm above the solution surface). The MO aqueous solution was magnetically stirred throughout the photocatalytic experiment to ensure the full suspension of particles. The experiments were conducted for 60 min with 3 mL sample aliquots extracted every 15 min and subsequently centrifuged at 4,000 rpm for 10 min. The decomposition of MO was monitored by measuring the absorbance of the supernatant at 464 nm using TU-2550 spectrophotometer.

## Results and discussion

### Electron microscopy studies

**Fig. 1:**
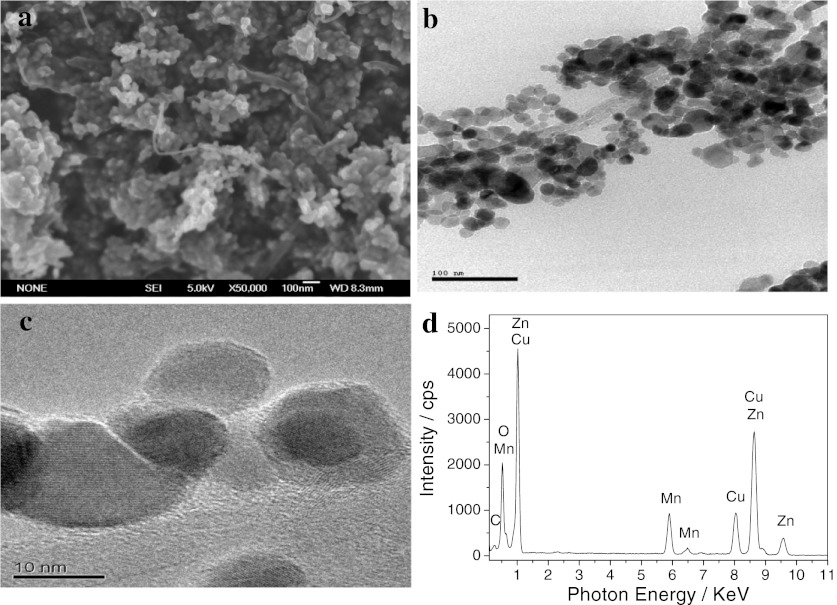
SEM (**a**), TEM (**b**), HRTEM (**c**) and EDS (**d**) of MWNT/Mn-doped ZnO hybrids

Figure 1 shows the SEM (a), TEM (b), HRTEM (c), and EDS (d) of MWNT/Mn-doped ZnO hybrids. Figure 1a (SEM image of MWNT/Mn-doped ZnO hybrids) displays that the surface of MWNTs is coated by nanoparticles. From TEM images of MWNT/Mn-doped ZnO hybrids, it is obvious that the MWNTs are uniformly modified by nanoparticles with sizes of the range 10–20 nm, as shown in Fig. 1b, c. In order to confirm the element present in sample, EDS is carried out, and the results are shown in Fig. 1d. It reveals the presence of Zn, Mn, Cu, O, and C. Because the peaks of element Cu are attributed to the Cu grid that supports the sample, we can deduce that the nanoparticles decorated on surfaces of MWNTs are Mn-doped ZnO nanoparticles.

**Fig. 2:**
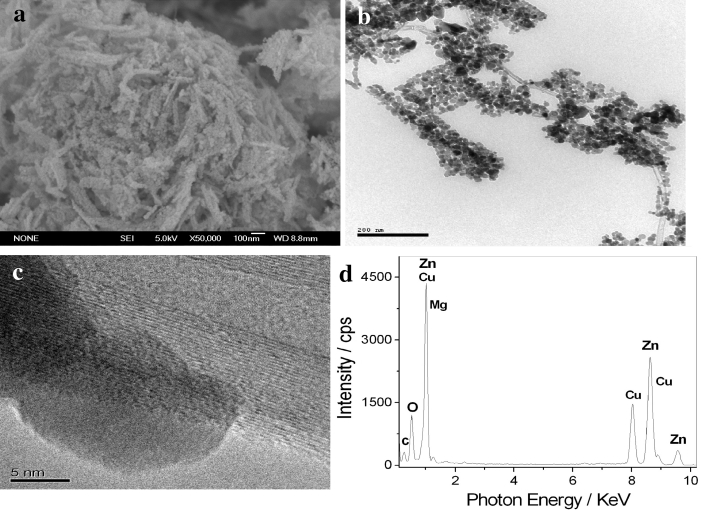
SEM (**a**), TEM (**b**), HRTEM (**c**) and EDS (**d**) of MWNT/Mg-doped ZnO hybrids

Figure 2 displays the SEM (a), TEM (b), HRTEM (c), and EDS (d) of MWNT/Mg-doped ZnO hybrids. Figure 2a shows SEM image of MWNT/Mg-doped ZnO hybrids. It is clear that MWNT/Mg-doped ZnO nanohybrids are composed of nanowires, and the size of the nanoparticles is about 10 nm. Further analysis of the MWNT/Mg-doped ZnO hybrids by TEM confirms that the MWNTs are uniformly decorated by nanoparticles with sizes of about 10 nm (shown in Fig. 2b, c). During the TEM measurements, the EDS analysis was performed. The EDS shows that the hybrids are mostly composed of Zn, Mg, O, and C elements, as shown in Fig. 2d. These results illustrate that MWNTs can be modified by Mg-doped ZnO nanoparticles.

**Fig. 3:**
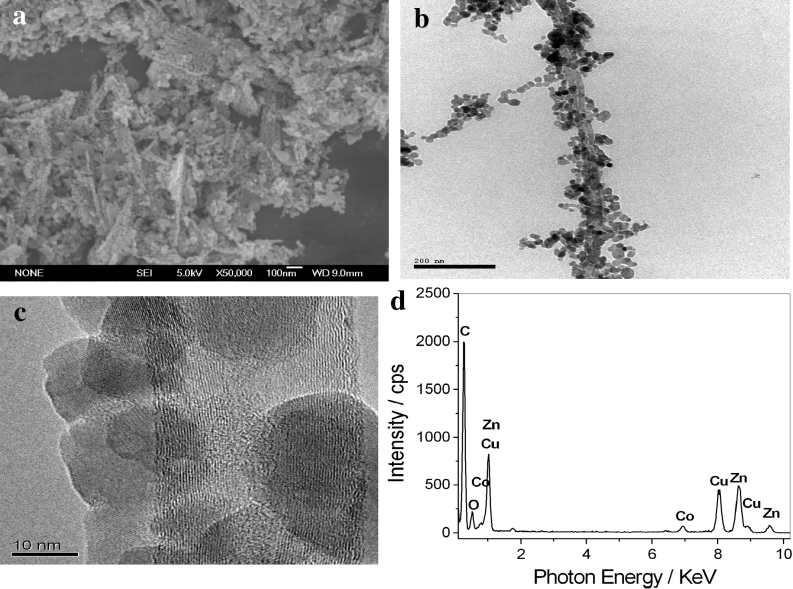
SEM (**a**), TEM (**b**), HRTEM (**c**), and EDS (**d**) of MWNT/Co-doped ZnO hybrids

Figure 3 is the SEM (a), TEM (b), HRTEM (c), and EDS (d) of MWNT/Co-doped ZnO hybrids. Figure 3a shows SEM image of MWNT/Co-doped ZnO hybrids. We can observe that the surface of MWNTs is decorated by a layer of nanoparticles. The TEM image (Fig. 3b, c) displays that MWNTs are uniformly modified by nanoparticles with sizes of about 10–20 nm. EDS analysis is carried out, and only the peaks of Zn, O, Co, and C element are observed in the EDS spectrogram of MWNT/Co-doped ZnO hybrids, as shown in Fig. 3d. These results indicate that the surfaces of MWNTs are decroated by Co-doped ZnO nanoparticles.

### XRD analysis

**Fig. 4:**
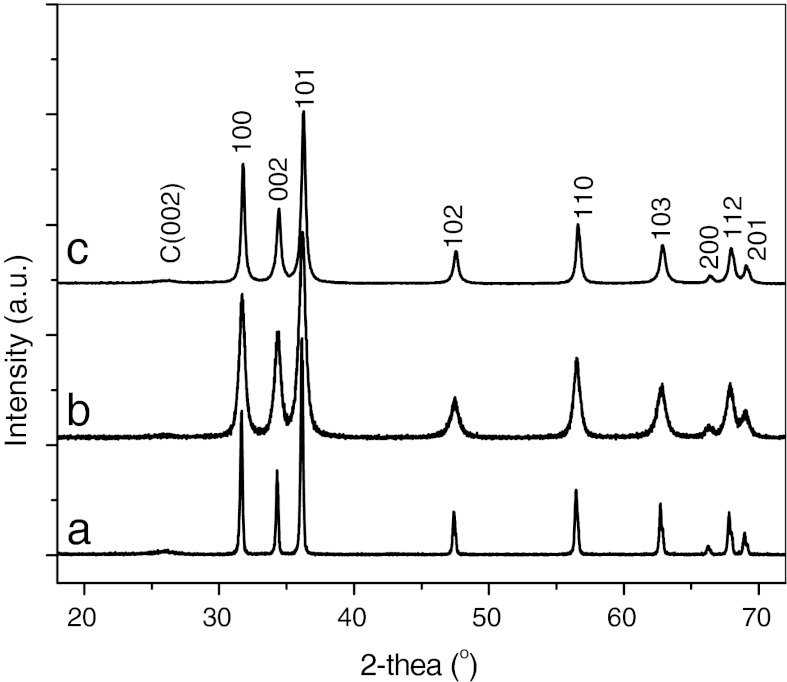
XRD of MWNT/metal-doped ZnO hybrids: *a* MWNT/Mn-doped ZnO hybrids, *b* MWNT/Mg-doped ZnO hybrids, and *c* MWNT/Co-doped ZnO hybrids

Powder XRD patterns were taken to examine the crystal structure of MWNT/metal-doped ZnO hybrids. Figure 4 shows the XRD of MWNT/metal-doped ZnO hybrids. XRD patterns of all samples exhibit nine diffraction peaks at 2θ = 31.86°, 34.45°, 36.32°, 47.72°, 56.70°, 62.94°, 66.65°, 68.03°, and 69.22°, respectively, pertaining to (100), (002), (101), (102), (110), (103), (200), (112), and (201) planes of ZnO. All diffraction peaks are in good agreement with those of the hexagonal wurtzite structure of ZnO (JCPDS card 36–1451). Concurrently, the (002) plane characteristic peak of graphite can be observed at 2θ = 26.16°. Although it was very weak, it confirms that the composite contains MWNTs. In addition, no trace of metal, oxide, or any binary zinc alloy phases are observed in the XRD pattern of all samples. This result indicates that doping metal element may replace the Zn site of ZnO lattice, or inserts into the crystal lattice of ZnO.

### Mechanism analysis of MWNT-supported nanoparticles

**Fig. 5:**
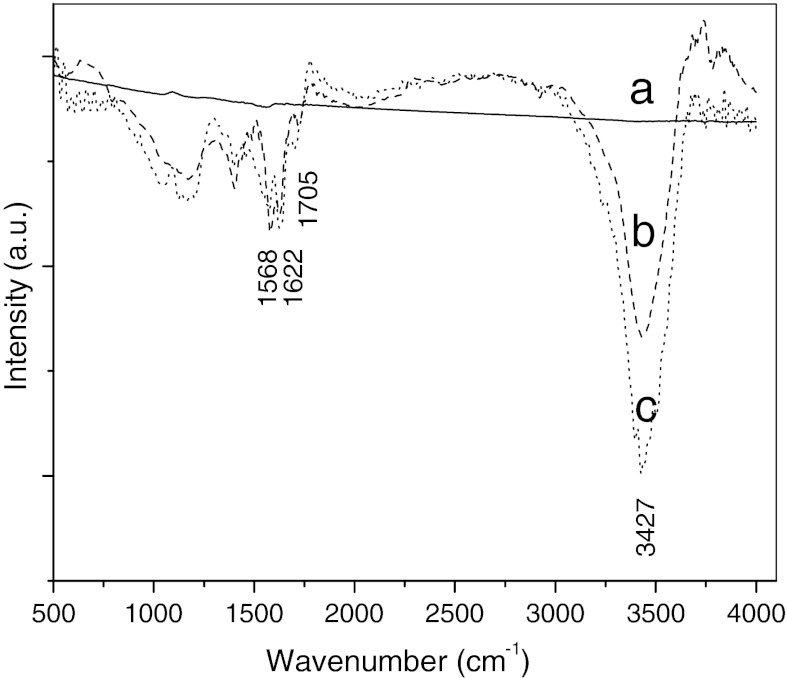
Infrared spectrum of different MWNTs: *a* as-prepared, *b* treated MWNTs by mixture acid, and *c* treated by NaOH and mixture acid

**Fig. 6:**
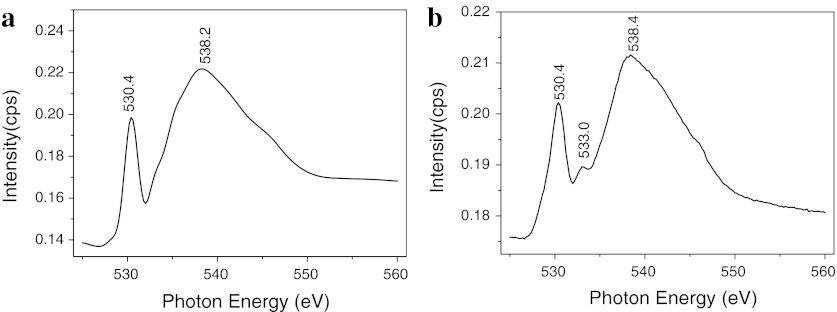
O *K*-edge XANES spectra of MWNTs: **a** as-prepared, **b** treated by NaOH and mixture acid

In order to discuss the mechanism-supported nanparticles on MWNTs, the MWNTs are characterized by infrared (IR) spectroscopy and SXMCD. Figure 5 shows the IR spectra of different MWNTs. It can be seen that no peaks are observed in the spectrum of pristine MWNTs (curve a in Fig. 5). However, after the MWNTs are treated, many groups are introduced onto the surface of MWNTs, such as carbonyl groups reveal at about 1,705 cm^−1^, and oxygen–hydrogen bonds and C=C bonds reveal at about 3,427 cm^−1^ and 1,568 cm^−1^, respectively, as shown in curve b and curve c of Fig. 5. Furthermore, we find that NaOH treatment enhances the intensity of oxygen–hydrogen bonds, which indicates that the surface activity of MWNTs is further improved. The O *K*-edge XANES spectra are used to test MWNTs, and the result is shown in Fig. 6. It is evident that all samples show two peaks at about 530.4 eV and 538.2 eV, respectively, which are attributed to physical absorption oxygen. Compared with Fig. 6a, b, we can observe a new peak at about 533.0 eV in the O *K*-edge XANES spectrum of treated MWNTs, resulting from the interaction of carbon and oxygen. In other words, the oxygen is adsorbed onto the surface of MWNTs by chemical interaction (Benndorf et al. 1996). This result further illustrates that the carbonyl groups are introduced onto the carbon atom of MWNTs surface after they are treated by NaOH and mixture acid.

**Fig. 7:**
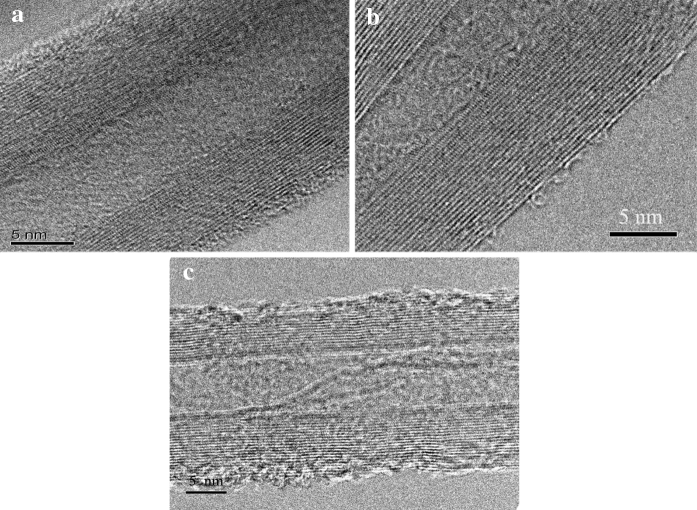
TEM image of different MWNTs: **a** as-prepared, **b** treated by mixture acid, and **c** treated by NaOH solution and mixture acid

Nitrogen adsorption–desorption isotherms were measured to determine the effect of surface treatment on the specific surface area of MWNTs. The results show that the specific surface area of as-prepared MWNTs is only 140.1 m^2^/g, and the specific surface area of MWNTs oxidized by mixture acid reach 186.2 m^2^/g. However, the specific surface area of MWNTs increases to 257.4 m^2^/g after being treated by NaOH solution and mixture acid. The improvement in specific surface area is because NaOH solution can etch the carbon atom of MWNTs surface, resulting in forming more holes on the wall of MWNTs (PIÑERO et al. 2005). Figure 7 displays the HRTEM image of different MWNTs. It can be seen that there is an amorphous layer on the outer layer of pristine MWNTs, as shown in Fig. 7a. After being treated by mixture acid, the amorphous layer is not observed on the outer surface of treated MWNTs (shown in Fig. 7b). It is worth noting that when the MWNTs are treated by NaOH solution and mixture acid, not only the amorphous layer is eliminated completely, but also some defects on the outer wall of MWNTs are present (Fig. 7c). This result illustrates that the walls of MWNTs treated by NaOH solution and mixture acid are etched more seriously than that of MWNTs oxidized by mixture acid.

We presume that the MWNT-supported metal-doped ZnO nanoparticles are attributed to the treatment of NaOH solution and mixture acid. Many oxygen functional groups are introduced onto the walls of MWNTs after being treated by NaOH solution and mixed acid, resulting in improving the chemical activity and dispersion of MWNTs. Moreover, these carboxyl groups on the wall of MWNTs can form carboxyl group with negatively charge in water solution, and easily attract positively charged metal ions in solution through electrostatic interactions, facilitating the interaction between MWNTs and zinc oxalate precursor (Eder 2010). In addition, NaOH solution can etch the carbon atom of MWNTs walls, and more defects are produced on the walls of MWNTs, which are also propitious to the deposition of nanoparticles on the surface of MWNTs. Hence, MWNTs can uniformly support metal-doped ZnO nanoparticles.

### Photocatalytic studies

Table 1 depicts the photocatalytic degradation efficiency of different samples for MO. The photocatalysts loading content is 0.25 g/L, and the initial concentration of MO is 20 mg/L. It is found that the blank test (without any sample) shows only a small amount of MO degradation, and the amount of MO degraded by the pure ZnO sample is 23.6 % after 60 min. However, the MWNT/ZnO hybrid sample exhibits higher photocatalytic degradation efficiency than pure ZnO sample, which comes up to 80.2 % in 60 min. In addition, the photocatalytic degradation efficiency of MWNT/Mn-doped ZnO sample and MWNT/Co-doped ZnO sample for MO is lower than that of MWNT/ZnO hybrid sample. It is worthy to note that MWNT/Mg-doped nanohybrids samples display very good photocatalytic degraded efficiency for MO, which the degraded rate for MO reaches about 100 % within 60 min. These results imply that the photocatalytic degraded ability of ZnO for MO is improved by MWNTs as well as Mg-doping.

**Table 1:** Photocatalytic performance of different samples for methyl orange

Samples	Degradation rate (%)			
Time (min):	15	30	45	60
Blank	2.4	5.3	7.6	10.9
ZnO	6.4	13.9	18.4	23.6
MWNT/ZnO hybrids	17.8	40.4	62.2	80.2
MWNT/Zn_0.95_Mn_0.05_O hybrids	8.9	16.8	22.9	27.6
MWNT/Zn_0.9_Mg_0.1_O hybrids	23.2	51.7	77.6	100
MWNT/Zn_0.9_Co_0.1_O hybrids	7.7	11.7	16.9	22.8

### Analysis of photocatalytic mechanism

**Fig. 8:**
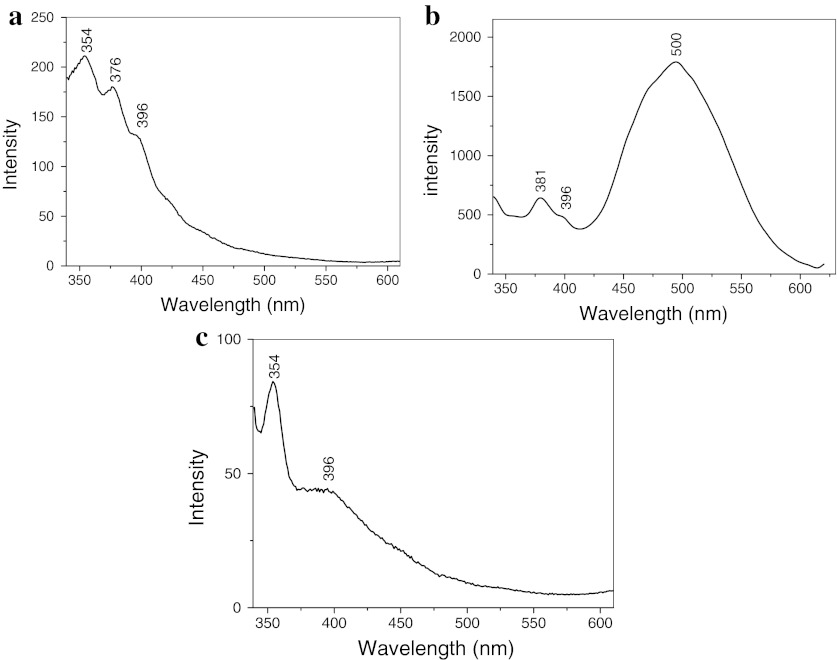
Fluorescence spectra of different samples: **a** MWNT/Zn_0.95_Mn_0.05_O hybrids, **b** MWNT/Zn_0.9_Mg_0.1_O hybrids, and **c** MWNT/Zn_0.9_Co_0.1_O hybrids

Figure 8 shows the room-temperature fluorescence spectrum of MWNT/metal-doped ZnO nanohybrids in which the wavelength of excitation is 320 nm. From the spectra of MWNT/Mn-doped ZnO hybrids and MWNT/Co-doped ZnO hybrids, we can see that there is a strong UV peak at about 354 nm, and the intrinsic peaks of ZnO at about and 376 and 396 nm, respectively. According to our previous reported results (Chen et al. 2012), the UV peak at about 354 nm comes from MWNTs. The peaks at about 376 and 396 nm are assigned to UV emission originating from the wide band gap of ZnO. However, in the spectrum of MWNT/Mg-doped ZnO nanohybrids, there is a very strong green emission band center at about 500 nm besides the intrinsic peaks of ZnO, which is attributed to the singly ionized oxygen vacancy in the ZnO nanostructures and the results from the recombination of electrons at the conduction band with holes trapped in oxygen-related defects (Fujihara et al. 2007). These results imply that Mg-doping can modulate the value of the bandgap of ZnO and increase the UV luminescence intensity.

It is concluded that the enhancement in photocatalytic activity is attributed to the excellent electriconal property of MWNTs and forming many defects in the ZnO crystals by Mg-doping. This is because MWNTs can capture the photon-excited electrons from the conduction band of the ZnO because of its large electron-storage capacity. Simultaneously, the captured photon-excited electron can be fast conducted by MWNTs, hindering the recombination of electron–hole pairs. In addition, when magnesium replaces the zinc site of ZnO lattice or inserts into the Zn interstitial, the lattice of ZnO is distorted and form oxygen vacancies and zinc vacancies, resulting in engendering intermediate energy gap between valence band and conduction band of ZnO (Li et al. 2009). The intermediate energy gap can promote the absorption of photons and create electron–hole pairs (Suwanboon Amornpitoksuk 2012). Hence, the photocatalytic property of ZnO is improved by Mg-doping significantly. However, the major emission peaks of MWNT/Zn_0.95_Mn_0.05_O hybrids and MWNT/Zn_0.9_Co_0.1_O hybrids were observed at about 354 nm, which is lower than the wavelength of excited lamphouse, resulting in the display of very low photocatalytic efficiency.

## Conclusion

In this article, we report a simple and versatile approach to decorate metal-doped ZnO on the surface of MWNTs through co-precipitation method. MWNTs can be modified by metal-doped ZnO nanoparticles when heat treatment is carried out at 450 °C. MWNTs can act as photosensitizers for ZnO nanoparticles and hinder the recombination of electron–hole pairs. Moreover, incorporating Mg into ZnO has been proven feasible to realize the band-gap modulation of ZnO, resulting in facilitating the generation of electron–hole pairs in ZnO crystals. Therefore, the synthetic MWNT/Zn_0.9_Mg_0.1_O hybrids display prominent photocatalytic activity, and can be utilized for photocatalysis, sewage treatment, and environmental protection.
